# Layered Potassium Titanium Niobate/Reduced Graphene Oxide Nanocomposite as a Potassium-Ion Battery Anode

**DOI:** 10.1007/s40820-023-01222-2

**Published:** 2023-11-06

**Authors:** Charlie A. F. Nason, Ajay Piriya Vijaya Kumar Saroja, Yi Lu, Runzhe Wei, Yupei Han, Yang Xu

**Affiliations:** Department of Chemistry, University College London, 20 Gordon Street, London, WC1H 0AJ UK

**Keywords:** Potassium-ion batteries, Intercalation, Transition metal oxides, Anodes, Nanocomposite

## Abstract

With graphite currently leading as the most viable anode for potassium-ion batteries (KIBs), other materials have been left relatively under-examined. Transition metal oxides are among these, with many positive attributes such as synthetic maturity, long-term cycling stability and fast redox kinetics. Therefore, to address this research deficiency we report herein a layered potassium titanium niobate KTiNbO_5_ (KTNO) and its rGO nanocomposite (KTNO/rGO) synthesised via solvothermal methods as a high-performance anode for KIBs. Through effective distribution across the electrically conductive rGO, the electrochemical performance of the KTNO nanoparticles was enhanced. The potassium storage performance of the KTNO/rGO was demonstrated by its first charge capacity of 128.1 mAh g^−1^ and reversible capacity of 97.5 mAh g^−1^ after 500 cycles at 20 mA g^−1^, retaining 76.1% of the initial capacity, with an exceptional rate performance of 54.2 mAh g^−1^ at 1 A g^−1^. Furthermore, to investigate the attributes of KTNO in-situ XRD was performed, indicating a low-strain material. Ex-situ X-ray photoelectron spectra further investigated the mechanism of charge storage, with the titanium showing greater redox reversibility than the niobium. This work suggests this low-strain nature is a highly advantageous property and well worth regarding KTNO as a promising anode for future high-performance KIBs.

**Supplementary Information:**

The online version contains supplementary material available at 10.1007/s40820-023-01222-2.

## Highlights

- KTiNbO_5_ and KTiNbO_5_/reduced graphene oxide (rGO) nanocomposites were successfully synthesised via solvothermal methods. Optimising the rGO wt% yielded a composite with 12 wt% (KTNO/rGO-12).
- KTNO/rGO-12 was tested for its potassium storage performance, achieving a first charge capacity of 128.1 mAh g^−1^ and retaining 76.1% over 500 cycles at 20 mAh g^−1^.
- The mechanism of intercalation was examined, suggesting a potentially low-strain material, with both titanium and niobium redox activity contributing to the charge storage.

**Supplementary Information:**

The online version contains supplementary material available at 10.1007/s40820-023-01222-2.

## Graphical abstract

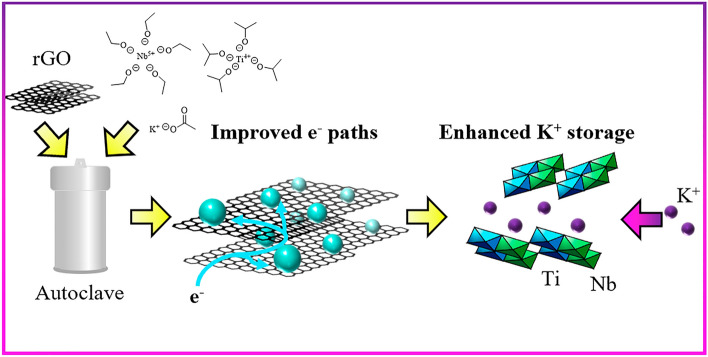

## Introduction

The anode of a rechargeable K-ion battery (KIB) has the key role of storing K^+^ de-intercalated from the cathode, with its performance affecting the capacity, charge/discharge rate and energy density of the cell. When considering KIBs, anodes receive a relatively lower proportion of research attention compared to cathodes and while there are many reasons for this, the main one is the success of graphite. Graphite has previously shone in Li-ion batteries (LIBs) as a low cost, high tap-density and high-capacity anode, with an advantageous plateau between 0.25 and 0.1 V versus Li/Li^+^ [1]. While potassium-graphite intercalation compounds like KC_8_ have been known since the 1960s, the electrochemical potassiation of graphite was only reported separately by Komaba et al. and Jian et al. in 2015, who elucidated the staging mechanism under electrochemical conditions [2–5]. The reported graphite anode achieved 244 mAh g^−1^ between 0 and 2 V versus K/K^+^ at 0.05 mV s^−1^, representing a performance milestone for carbon-based anodes. This was quickly followed by an explosion of investigation into graphite and carbon-based anode materials including hard/soft carbon, hard carbon-soft carbon composites and heteroatom doped carbons [6–10]. Graphite remains the most viable, as many of the alternative carbonaceous materials have sloping charge–discharge curves, which result in lower energy density and more complicated component design to compensate for the varying voltage. Despite graphite’s benefits, it has shortcomings, as it suffers from limited rate capability, poor cycling stability and significant solid-electrolyte interphase (SEI) formation in traditional carbonate electrolytes [11]. In addition, despite the advantage from an energy density perspective, the low voltage plateau at which K^+^ intercalates into the graphite is close to the K plating potential, which is a possible safety concern, reducing its viability.

To supplement graphite’s shortcomings, other electrode materials have been investigated. These fall into the categories of alloying, conversion, and intercalation compounds. Alloying and conversion compounds, while having exceptionally high capacities compared to intercalation anodes [12], often show extreme volume changes (> 300%) [13–15], causing electrode degradation and poor cyclability. Consequently, current commercial efforts are focused on intercalation-based anodes [16, 17]. Amongst the numerous materials reported, transition metal oxides (TMOs) are well regarded as promising candidates, with the focus on titanium (Ti), niobium (Nb) and molybendum (Mo) as the redox active metals. Of these, Ti and Nb are more promising, due to their higher natural crustal abundance of 5650 and 20 ppm, respectively, compared to the abundance of Mo (1.2 ppm) [18]. With significant synthesis maturity, TMOs can be tailored for specific applications to a far greater degree than graphitic anodes, with many morphologies and phases to suit the end use case. In addition, as intercalation anodes show small volume changes < 10% [17], continual SEI formation during cycling can be very limited, resulting in enhanced cycling stability in comparison to graphite and conversion/alloying compounds. TMOs have been shown to have high energy density, desirable voltage profiles and excellent stability, with materials such as Nb_2_O_5_, K_2_Ti_8_O_17_, K_2_Ti_2_O_5_, K_2_Ti_4_O_9_ and TiNb_24_O_62_ all showing promising initial results [19–25]. However, there is currently a significant research deficiency in the usage of TMOs as intercalation anode for KIBs. To the best of the authors’ knowledge, there has been only less than a dozen research publications reporting TMOs as intercalation KIB anodes so far [20–24, 26–29]. Therefore, any further investigation into TMOs would be valuable in addressing this deficiency.

**Fig. 1:**
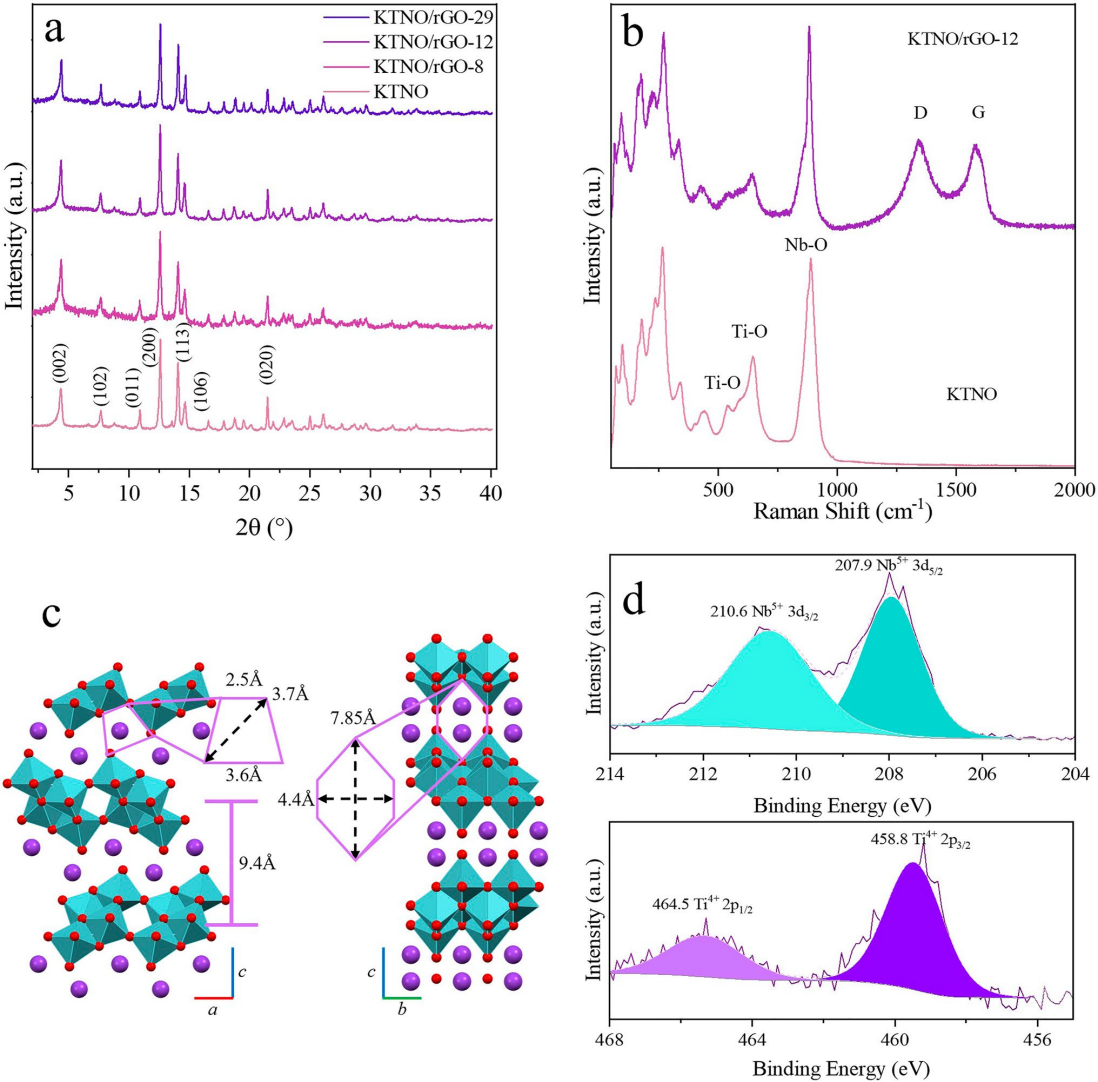
Materials characterisation of KTNO/rGO-12 and KTNO. **a** XRD pattern of KTNO and KTNO/rGO nanocomposites KTNO/rGO-8, KTNO/rGO-12 and KTNO/rGO-29. **b** Raman spectrum of KTNO and KTNO/rGO-12. **c** Crystal structure of KTNO, with diffusion channels indicated. **d** High resolution XPS spectra of Ti and Nb in KTNO/rGO-12

KTiNbO_5_ (KTNO) combines many of the positive traits that are desirable in intercalation anodes. The structure of KTiNbO_5_ was elucidated by Wadsley in 1963 and can be described as an infinite layer of double zigzag, corner and edge-sharing MO_6_ octahedra with K^+^ ions in-between the layers [30]. This zigzag structure arises from distortions in the MO_6_ octahedra, with M–O bond lengths ranging from 2.309 to 1.696 Å, creating puckered sheets. This is a common structure for KIB intercalation type transition metal oxide anodes, as K_2_Ti_4_O_9_, K_2_Ti_6_O_13_ and K_2_Ti_8_O_17_ all share this pattern [20, 23, 24, 26], with slight variations. This two-dimensional (2D) corrugated structure forms channels along the (200) and (020) planes, facilitating K^+^ intercalation (Fig. 1c). It additionally has a particularly large *d*-spacing of 0.94 nm allowing for the easy intercalation of charge carrying species [31, 32]. This large *d* spacing is primarily the reason for the use of KTNO in previous work, as it has been ion-exchanged with H^+^ to boost storage in LIBs and Na-ion batteries (NIBs), or to exfoliate it into TNO nanosheets [28, 33, 34]. KTNO has been tested as a baseline material, but no work has focused on it as a primary material. In this work, we report the synthesis, performance and mechanism of KTNO/reduced graphene oxide (rGO) nanocomposite as an intercalation-based anode material. By controlling the size of the nanoparticles via synthetic methods, KTNO can be improved to a viable intercalation anode for KIBs. The performance can also be further improved via the inclusion of rGO, to form a KTNO/rGO nanocomposite. This has several benefits. The poor inherent electronic conductivity of KTNO can be partially mitigated by the dispersion of the oxide nanoparticles across the rGO sheets, with the sheets acting as a conductive matrix for electrons to reach the oxide particles [35, 36]. This in-situ approach also greatly promotes nucleation and improves coverage of rGO by the nanoparticles, nucleating on the rGO surface, as the remaining surface oxygen functional groups allow for anchoring of the metal centres [37]. The KTNO nanoparticles can also prevent rGO from restacking, possibly synergistically improving the cyclability of the nanocomposite [38, 39]. Amongst the three optimised KTNO/rGO nanocomposites, 12 wt% rGO represents the optimised performance with relation to the rGO content, achieving 128.1 mAh g^−1^ at 20 mA g^−1^ on the first charge, along with retaining 76.1% of its initial charge capacity over 500 cycles. This is among the best performing of the TMO intercalation anodes reported so far, rivalling other cutting edge TMO materials and indicating an exciting new area of study into similar structure types for KIBs.

## Experimental Section

### Preparation of KTNO/rGO Nanocomposites

KTNO was prepared via solvothermal methods [33]. Titanium isopropoxide (1.1 mmol, 99.999%, Sigma Aldrich), niobium ethoxide (1 mmol, 99.8%, Sigma Aldrich) and potassium acetate (5.7 mmol, 99%, Sigma Aldrich) were added to 2-propanol in (IPA, 30 mL, Reagent-Grade) and then stirred for 5 min. Ethylenediamine (EDA, 200 µL, 1.8 mmol) was then added, and the solution was stirred for 5 h. The KTNO solution was then transferred to a Teflon-lined autoclave (Parr, 66.6% fill factor) and kept at 200 °C (5 °C min^−1^ ramp rate) for 12.5 h. The resulting white precipitate was separated by centrifuge (4500 rpm, 15 min) and washed with deionised water and ethanol for three times, then vacuum dried at 60 °C overnight. The resulting solid was ground via mortar and pestle, then calcinated in air at 700, 900 and 1000 °C for 5 or 10 h (5 °C min^−1^ ramp rate). rGO was prepared via Hummer’s method [40], then calcinated at 300 °C for 3 h in 5% H_2_/N_2_ [40]. The KTNO/rGO nanocomposite was prepared as above by adding various amount of rGO (10, 15, 30 wt% estimated on 100% predicted yield of KTNO) to the initial solution pre-autoclaving and pre-stirring, yielding a KTNO/rGO nanocomposite after calcinating in N_2_ at 700 °C for 5 h.

### Materials Characterisation

Powder X-ray diffraction (XRD) and in-operando XRD were carried out on a Stoe STADI-P diffractometer using Mo Kα1 radiation (0.709 Å, 50 kV, 30 mA) at a scan rate of 30 s degree^−1^, between 2.000° and 40.115° or 32.185°. Thermal gravimetric analysis (TGA) analysis was performed on a PerkinElmer Simultaneous Thermal Analyser (STA) 6000 (20 mL min^−1^, 800 °C, 10 °C min^−1^). Scanning electron microscopy (SEM) analysis was performed on Jeol JSM 6701 Field Emission Gun—Scanning Electron Microscope, with energy-dispersive X-ray spectroscopy (EDS) analysis collected on Jeol JSM 7600 Field Emission Gun—Scanning Electron Microscope. Transition electron microscopy (TEM) analysis was performed on Jeol 2100 transmission electron microscope. X-ray photoelectron spectra (XPS) were recorded on ThermoScientific Kα X-Ray Photoelectron Spectrometer, with charging correction applied using C 1*s* as a reference at 285.00 eV.

### Electrochemical Measurements

Electrode slurries were prepared in a 75:15:10 wt% ratio of active material:Super P:carboxymethylcellulose (CMC), which was then coated via the doctor blade technique onto copper foil, then dried under vacuum at 90 °C for 12 h. Mass loadings of active material on 16 mm diameter electrode disks were between 0.5 and 2 mg cm^−2^ for all electrochemical analysis. CR2032 stainless steel coin cells were assembled in an Ar filled glovebox (O_2_ and H_2_O < 0.1 ppm) with K metal as the counter electrode, glass microfibre (Whatman, Grade B) as the separator and 1 M potassium bis(trifluoromethanesulfonyl)imide (KFSI) in the mixture of ethylene carbonate (EC) and diethylene carbonate (DEC) (1:1, v:v) as the electrolyte. Cyclic voltammetry (CV) and electrochemical impedance spectroscopy (EIS) were measured on a Gamry Interface 1010E potentiostat (0.001–3 V vs. K/K^+^). Galvanostatic charge–discharge (GCD) curves were measured on Neware battery cyclers (0.001–3 V vs. K/K^+^). The in-operando coin cell was prepared using a Kapton windowed CR2032 stainless steel coin cell, with a window punched in both the potassium counter electrode and the separator, exchanging the stainless steel spacer for a 0.4 mm thick aluminium spacer. For GITT analysis, 20/30-min pulses of 20 mA g^−1^, followed by a rest of 2 h.

## Results and Discussion

### Physical Characteristics of KTNO and KTNO/rGO Nanocomposite

The KTNO/rGO nanocomposite was successfully synthesised with the addition of rGO in the first step pre-autoclaving. The weight percent (wt%) of rGO was varied to optimise electrochemical performance while retaining the maximum wt% of KTNO as possible. Tested via thermogravimetric analysis (TGA) the wt% of rGO in the synthesised nanocomposites was determined to be 7.5%, 12.3% and 29.2%, respectively (Fig. S1). They will therefore be denominated as KTNO/rGO-8, KTNO/rGO-12 and KTNO/rGO-29. The rGO sheets used to synthesise the nanocomposites displayed a crumpled morphology, with the characteristic GO peak disappearing from the XRD pattern upon reduction (Figs. S2, S3). The survey and high-resolution XPS spectra also indicate the GO was reduced by the H_2_/N_2_ (Figs. S4, S5), with some O functional groups remaining to interact with the KTNO nanoparticles.

Figure 1a shows the XRD patterns of KTNO and the KTNO/rGO nanocomposites, all of which were matched to KTNO (ICSD: 98-000-3227), indicating the purity of the samples. The presence of carbon in the nanocomposite was confirmed via Raman spectroscopy (Fig. 1b), where peaks at 539 and 646 cm^−1^ can be attributed to corner-sharing and edge-sharing TiO_6_ octahedra, respectively, with the peak at 891 cm^−1^ attributable to corner-sharing NbO_6_ octahedra [41–44]. In addition, two peaks were observed at 1341 and 1586 cm^−1^, corresponding to the D and G bands of carbon, respectively, with an obtained I_D_/I_G_ ratio of 1.05, calculated via absolute intensities. For KTNO/rGO-12, the Ti core level XPS spectrum can be assigned to Ti 4 + , with components at 464.5 and 458.8 eV (Fig. 1d) due to the split of 2p_1/2_ and 2p_3/2_, respectively [45]. The Nb can also be assigned in its 5 + oxidation state, with peaks at 210.6 and 207.9 eV due to the split of 3d_3/2_ and 3d_5/2_, respectively [45]. This indicates that the active redox centres of the Ti and Nb were unchanged in the synthesis of the nanocomposite.

**Fig. 2:**
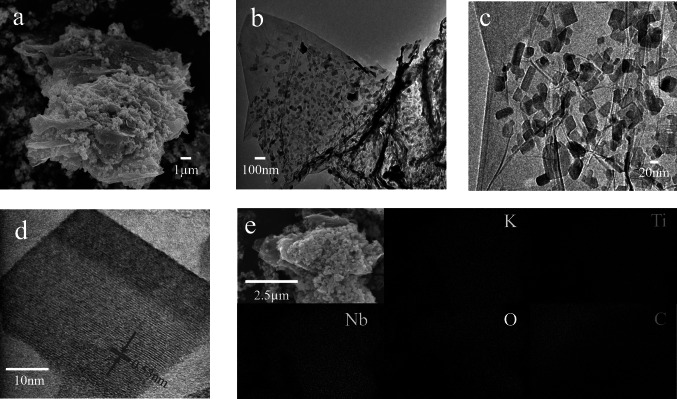
Morphology and elemental distribution of KTNO/rGO-12. **a** SEM image of KTNO/rGO-12. **b** TEM image of KTNO/rGO-12. **c** HRTEM image of KTNO particle morphology. **d** HRTEM image of KTNO/rGO-12 with (102) lattice plane of 0.53 nm indicated.** e** EDS elemental mapping of KTNO/rGO-12

SEM and TEM analysis revealed the surface of rGO was very well covered by the nanoparticles (Fig. 2a–c). The TEM images clearly show the thin nature of rGO with wrinkles and the dispersion of the KTNO particles on the surface of rGO. The particles are largely dispersed homogeneously, but there are areas that have observably more KTNO than other areas (Fig. S6). This is due to the limited solubility of rGO in IPA, rendering rGO unevenly distributed in the solution, leading to a gradient of coverage [46]. This was seen with all the nanocomposites but particularly with the 30 wt% sample. The morphology of rGO was unchanged during nanocomposite synthesis. The characteristic (102) lattice fringes of KTNO in KTNO/rGO-12 were observed via HRTEM (Fig. 2d), showing the presence and high crystallinity of the KTNO nanoparticles distributed on rGO, which confirms the inclusion of rGO has no impact on the crystallinity of the nanoparticles. EDS mapping supported the XPS results that the nanoparticles contained a homogenous distribution of K, Ti, Nb with the atomic ratio of 1:0.86:1.07 (Figs. 2e and S7, S8).

KTNO was also successfully synthesised and well indexed to orthorhombic KTiNbO_5_ (ICSD: 98-000-3227) with the space group of Pnma (Figs. 1a and S9) [47]. The (002) peak at 4.339°, corresponds to a large interlayer spacing of 9.4 Å. During preliminary synthesis, minor phases of the impurity KNbO_3_ were observed. This was traced back to EDA, which has been known to chelate strongly to Ti^4+^, thus removing it from the initial composite forming reaction and resulting in the formation of KNbO_3_ [48–50]. To reduce this phase while still retaining the reducing and chelating effects of EDA, the concentration was kept low, with an excess of Ti precursor. For KTNO, the nanoparticles were 100–200 nm in size, with an irregular morphology depending on the calcination temperature (Fig. S10). At 900 and 1000 °C, the quantity and size of these plates increased significantly, with the particle sizes increasing to > 1 µm, thus allowing for the control over the size of the KTNO particles (Fig. S10). Previous work has also recorded this calcination temperature to morphology and size dependence, which is due to the preferential growth in the (002) plane at higher temperatures, leading to nanorods and nanoplates [33, 51]. Due to the lack of adequate ionic channels in the *c* direction, growth along this axis is not conducive to assisting intercalation. So, to retain the optimum particle morphology, the temperature and time of calcination were tightly controlled, resulting in the optimised calcination temperature of 700 °C. EDS elemental distribution mapping on KTNO was performed, (Fig. S11) indicating that there was a homogenous distribution of K, Ti, Nb and O. The TEM image of KTNO (Fig. S12) reveals lattice fringes of 0.94 nm, the (002) plane as expected, and confirms the crystallinity of the nanoparticles. The chemical composition and oxidation states of KTNO were investigated via XPS analysis. As shown by the survey spectrum (Fig. S13), the ratio of K, Ti and Nb is within the values expected, giving a 1:1.06:1.05 ratio. To investigate the bonding environment of the redox active species, Ti and Nb, high resolution spectra were taken (Fig. S14). In the Ti spectrum, the 2p_1/2_ and 2p_3/2_ peaks, at 464.5 and 458.8 eV, respectively, match well to the literature values for Ti^4+^ [45]. For Nb, two peaks were observed with positions of 209.9 and 207.2 eV matching literature values for Nb^5+^, indicating that Ti and Nb are in their highest oxidation state [45].

### Potassium Storage Performance of KTNO and KTNO/rGO Nanocomposite

**Fig. 3:**
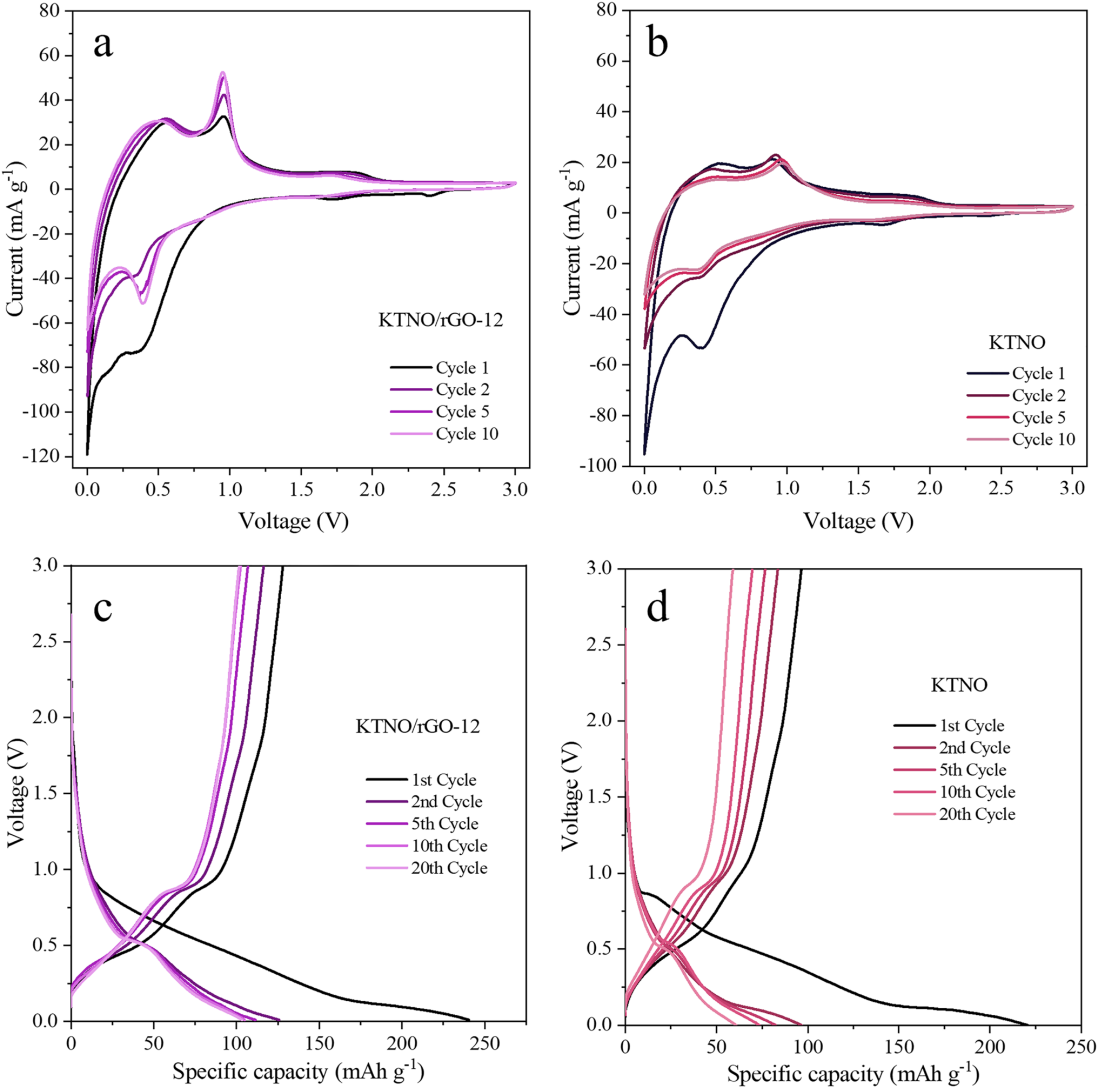
Electrochemical performance of KTNO/rGO-12 and KTNO. **a, b** CV curves of KTNO and KTNO/rGO-12 at 0.1 mV s^−1^. **c, d** Galvanostatic charge–discharge curves of KTNO and KTNO/rGO-12 at 20 mA g^−1^

For analysing the potassium storage properties of the KTNO/rGO nanocomposites, a potential window of 0.001 to 3 V was chosen, as the redox potentials of Nb^5+^/Nb^4+^, Nb^4+^/Nb^3+^ and Ti^4+^/Ti^3+^ are in the anode range. Using a scan rate of 0.1 mV s^−1^, the CV curves of KTNO/rGO and KTNO were measured (Figs. 3a, b and S17). For both KNTO/rGO-12 and KTNO two symmetric peaks were recorded at 0.9 V on the anodic scan and 0.4 V on the cathodic scan, along with two smaller peaks at 1.91 and 1.77 V on the anodic and cathodic scan, respectively. Ti reduction has been observed to occur over a wide potential range in literature, giving rise to sloping charge–discharge curves with no obvious plateaus [20, 21, 23, 24]. The smaller peaks at 1.91 and 1.77 V can therefore be assigned to the start of the Ti^4+^/Ti^3+^ reduction. The peak at 0.4 V on the cathodic scan likely originates from both the continued Ti^4+^/Ti^3+^ reduction and the Nb^5+^/Nb^4+^ redox pair at a lower voltage [21–23, 52–54]. Combining literature results with our XPS analysis (Fig. 6b) leads to the assignment of the main peak at 0.4/0.9 V to be a contribution of both Ti^4+^/Ti^3+^ and Nb^5+^/Nb^4+^, but with the Ti^4+^/Ti^3+^ reduction process starting much earlier, seen by the 1.91/1.77 V peak. Ti is then reduced over a wide voltage range, contributing to the sloping curve between 1.77 and 0.4 V, whereas Nb is reduced around 0.4 V, giving rise to the major peak as both metals are contributing to the observed current.

For KTNO, the major anodic peak shifted from 0.9 to 0.97 V over 10 scans due to polarization of charge storage in the material. The major cathodic peak remained consistently at 0.4 V with little shifting over the 10 cycles. This shifting was not observed for the nanocomposites, indicating that rGO was assisting the conductivity, reducing the polarisation of the material, but providing a large current contribution, as seen from the broad peak at 0.5 V on the anodic scan and the long tail at close to 0 V on the cathodic scan (Fig. S18). The high reversibility combined with symmetric peak shapes gives evidence for a K^+^ intercalation/de-intercalation mechanism, but the exact redox pairs cannot be determined from CV alone. For the nanocomposite, the current response was enhanced, with the KTNO/rGO-12 achieving an *i*_*p*_ of 52.6 mA g^−1^, compared to KTNO, which displayed a 27.1 mA g^−1^ response, indicating an increase in capacity as rGO is acting as an electrically conductive network with the KTNO particles distributed over it.

For the K^+^ storage performance of the materials, within the 0.001–3 V window, KTNO/rGO-12 achieved a first charge of 128.1 mAh g^−1^ and a second discharge capacity of 125.7 mAh g^−1^ and an initial coluombic efficiency (ICE) of 53.3%, at 20 mA g^−1^ (Fig. 3c). This is 41% of the theoretical capacity of 309 mAh g^−1^, assuming a three-electron transfer with Nb^5+^/Nb^3+^ and Ti^4+^/ Ti^3+^. However, if considering a two-electron transfer, excluding Nb^3+^, then the theoretical capacity is 206 mAh g^−1^, a 62% utilisation. This exclusion of Nb^3+^ is reasonable due to the scarcity of reports for Nb^3+^ within KIBs, indicating that Nb^4+^/Nb^3+^ is a potentially difficult redox pair to activate. KTNO/rGO-8 and KTNO/rGO-29 additionally displayed a first charge of 120.0 and 143.0 mAh g^−1^ and a second discharge capacity of 119.0 and 145.1 mAh g^−1^ resulting in an ICE of 47.7% and 49.5% respectively (Fig. S19). KTNO on the other hand, achieved a first charge capacity of 96.7 mAh g^−1^ and a second discharge capacity of 96.6 mAh g^−1^ (Fig. 3d). By 20 cycles this had dropped to 60.6 mAh g^−1^, a 66.9% drop. The capacity is also higher than previously reported (50 mAh g^−1^) and is believed that this increase is due to the nanoparticle morphology, as the number of available ionic channels is drastically increased, resulting in greater intercalation and reduced polarization [34, 55]. However, this increase in surface area also results in more irreversible processes like side reactions with the electrolyte, forming more SEI, as noted from the low initial Coulombic efficiency (ICE) of 39%. This irreversible capacity loss can be attributed to both the small particle size that increases SEI generation and the irreversibility of the redox processes governing the potassiation/de-potassiation process [56, 57]. However, comparing the ICE of KTNO and KTNO/rGO nanocomposites to previous works studying intercalation-based transition metal oxide anodes, the initial CE in this work is among the best ones [20, 21, 23, 26]. After 20 cycles, coulombic efficiency (CE) for KTNO rose to 97.4%, suggesting high reversibility of intercalation and little structural breakdown. The CE further rose to 98% by 45 cycles and remained consistent after that. The passivation of the nanocomposite was shown to be enhanced when compared to the KTNO, as KTNO/rGO-12 reached a CE of 98.3% by cycle 11, which is earlier than KTNO. Notably there is a small plateau at 0.4 V in the discharge cycle and 0.9 V in the charge cycle of all KTNO materials tested, matching the CV peaks well, which gives further evidence of an intercalation style storage mechanism. This plateau has not been reported for KTNO previously.

**Fig. 4:**
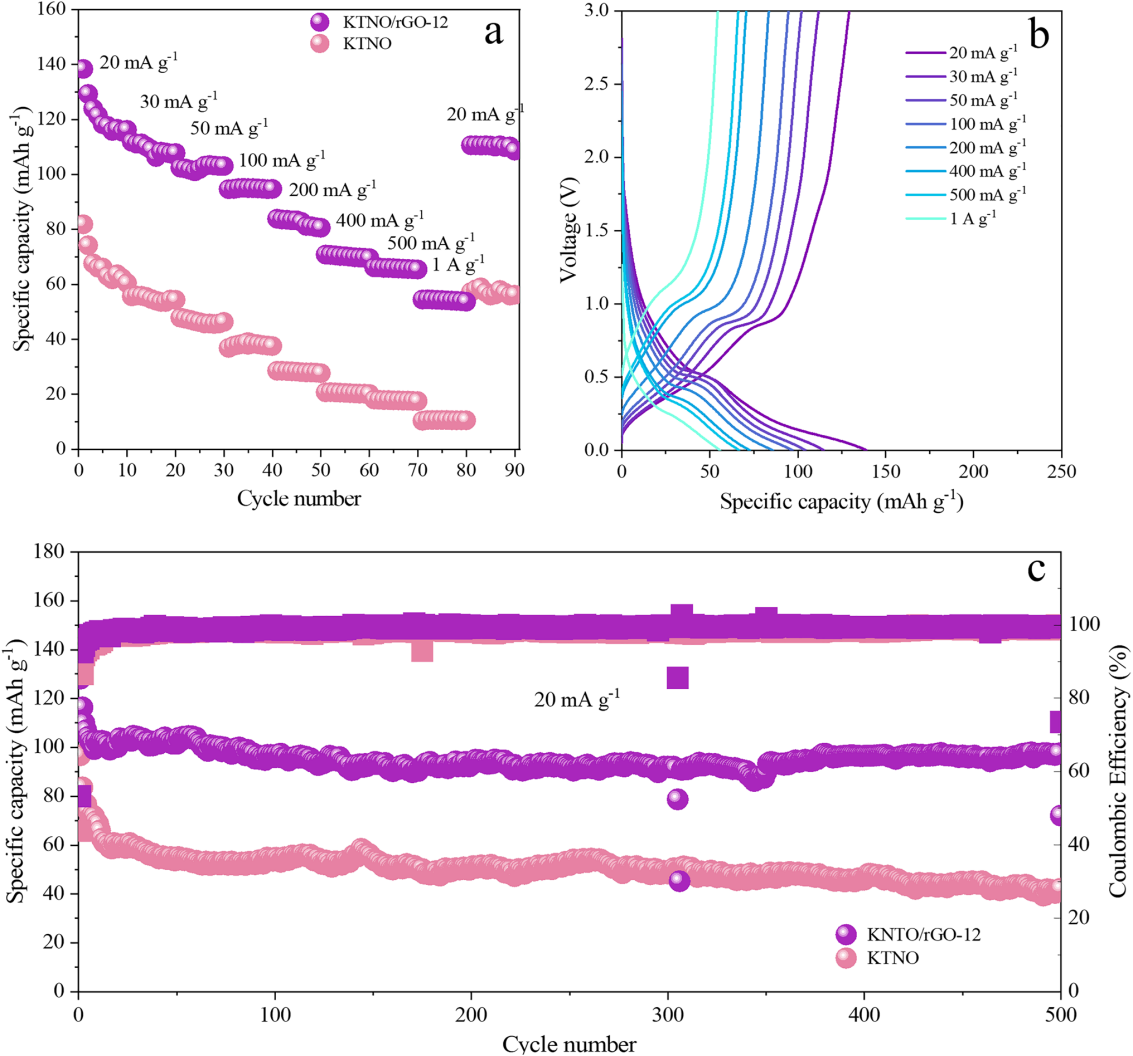
Rate and cycling performance of KTNO/rGO-12 and KTNO. **a** Rate performance comparison of KTNO and KTNO/rGO-12 between 20 mA g^−1^ and 1 A g^−1^. **b** Galvanostatic charge–discharge curves of KTNO/rGO-12 between 20 mA g^−1^ and 1 A g^−1^. **c** Long term cycling performance of KTNO and KTNO/rGO-12 at 20 mA g^−1^ over 500 cycles

The rate performance of KTNO/rGO and KTNO was tested from 20 mA g^−1^ to 1 A g^−1^ using charge capacity (Fig. 4a). With increasing the current density, KTNO/rGO-12 achieved a reversible charge capacity of 94.8 mAh g^−1^ at 100 mA g^−1^ and 54.2 mAh g^−1^ at 1 A g^−1^, amongst the highest reported rate performance in the KIB intercalation anode literature (Fig. 4a, b) [22, 58]. When comparing the pristine material to the nanocomposite, the addition of rGO clearly improved the rate performance, as charge capacity for KTNO dropped by 75%, from 66.7 mAh g^−1^ at 20 mA g^−1^ to 10.5 mAh g^−1^ at 1 A g^−1^, although still showing good rate performance when compared to previous reports [20, 53]. Comparing to the other nanocomposites, this increase was not dependant on the amount of rGO, as increasing the ratio from 8 to 29 wt% had no effect on the capacity at very high rates of 1 A g^−1^ (Fig. S20). This indicates a synergistic effect with rGO and KTNO, which is not a simple linear relationship, i.e., a higher rGO content not resulting in better rate capability. This is in line with the previous CV results, as the KTNO/rGO-12 showed the best current response at the prominent redox peaks. This indicates that 12 wt% is a good compromise between capacity, rate performance and stability. The rGO alone performed comparable to previous reports (Figs. S21 and S22). The long-term stability of KTNO/rGO and KTNO was investigated at low current density of 20 mA g^−1^ (Fig. 4c). The KTNO/rGO-12 retained 97.5 mAh g^−1^ (76.1%) of capacity over 500 cycles, compared to the 42.1 mAh g^−1^ (43.5%) for KTNO, which is excellent stability for both and is among the most stable materials in the KIB intercalation anode literature [53]. KTNO/rGO-8 and KTNO/rGO-29 retained 64.6 (53.8%) and 91.0 mAh g^−1^ (63%), respectively (Fig. S23). Due to these results, KTNO/rGO-12 was considered the overall optimised product, as it displayed excellent capacity, rate capability and stability, while containing a low wt% of rGO, so electrochemical analysis was focused on this sample.

### Electrochemical and Mechanistic Analysis of KTNO and KTNO/rGO Nanocomposite

**Fig. 5:**
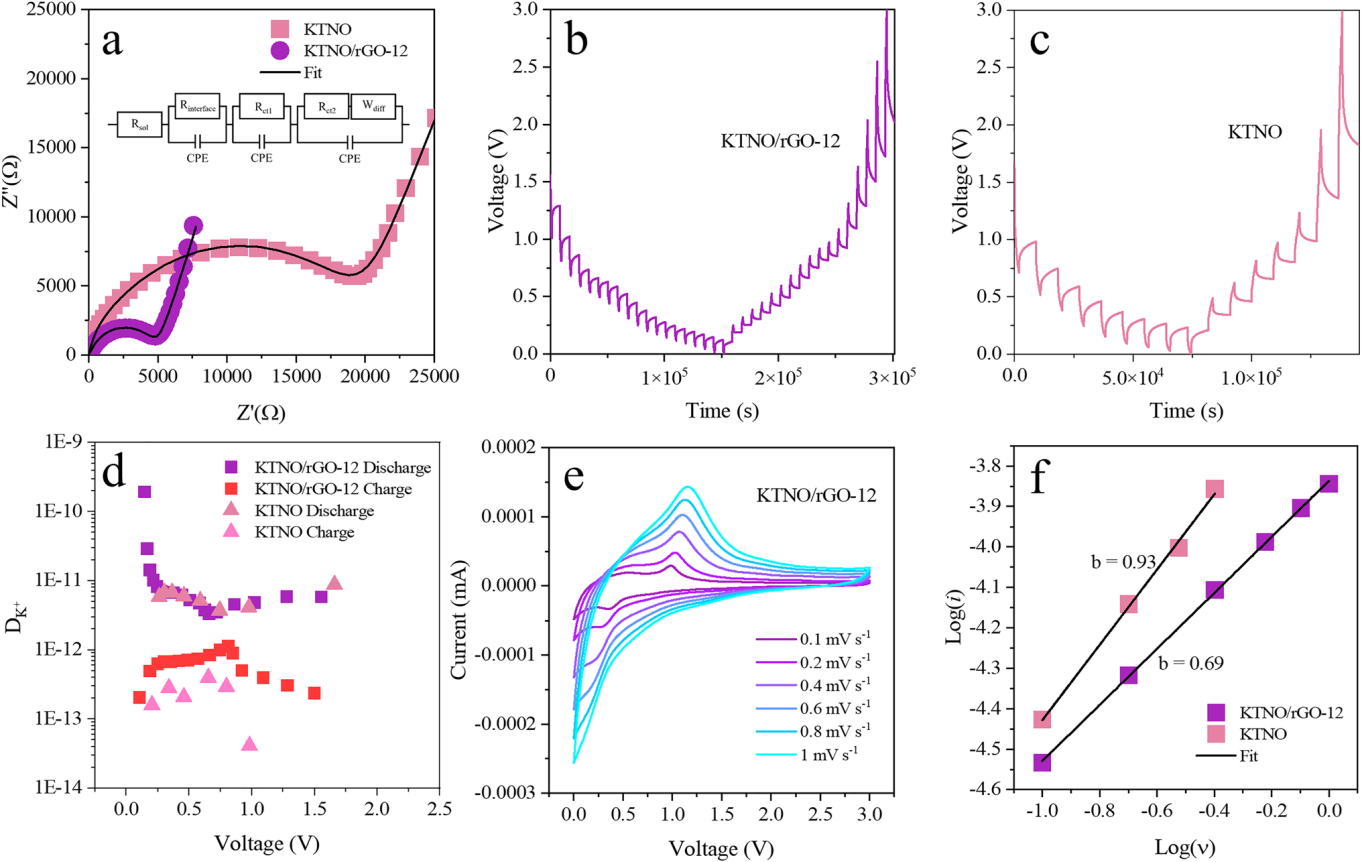
Electrochemical kinetics analysis of KTNO/rGO-12 and KTNO.** a** Nyquist plots of KTNO/rGO-12 and KTNO at OCP, with equivalent circuit as inset. **b** GITT potential profile of KTNO/rGO-12. **c** GITT potential profile of KTNO. **d** Corresponding D_K_^+^ values** e** CV curves of KTNO/rGO-12 at scan rates from 0.1 to 1 mV s^−1^. **f** Relationship between the scan rate and the peak current of KTNO/rGO-12 and KTNO

To investigate the internal resistances within the nanocomposite, EIS measurements were performed (Figs. 5a and S24) and analysed with the equivalent circuit displayed in Fig. S25. The equivalent circuit for all materials (Fig. 5a) contains components labelled *R*_*sol*_, *R*_*interface*_, *R*_*ct1*_ and *R*_*ct2*_ that represent the ohmic resistance for the electrolyte, the SEI, the carbon-based materials and the KTNO, respectively. These are paired with constant phase elements (*CPE*) to adjust for the non-ideal capacitor-like nature of these surfaces, along with a Warburg element (*W*_*diff*_) to simulate the semi-infinite linear diffusion of K^+^ within the KTNO lattice [59]. As expected, the charge-transfer resistance (*R*_*ct*_) of KTNO/rGO-12 was significantly lower than KTNO, at 4.6 and 16.9 kΩ, respectively. This reduction likely originates in the improved electron paths through the conductive rGO sheets, which in turn enhanced ionic conductivity of KTNO, resulting in improved electrochemical performance. To further demonstrate this improved electrochemical performance, galvanostatic intermittent titration technique (GITT) was performed to estimate the diffusion coefficient of K^+^ (*D*_*k*_) within the nanocomposite (Fig. 4b, c). The *D*_*k*_ values for KTNO/rGO-12 are calculated (Eq. S1) to be between 1.93 × 10^–10^ and 2.03 × 10^–13^ cm^2^ s^−1^, compared to 8.7 × 10^–12^ and 4.08 × 10^–14^ cm^2^ s^−1^ for KTNO (Fig. 5d). The higher *D*_*k*_ values for KTNO/rGO-12 indicate the presence of rGO assists the diffusion of K^+^ in the structure, even at relativity low amounts, 12 wt% [60]. By collecting CV curves at varying rates, the balance between capacitive and diffusion dominated processes can be deconvoluted, with CVs of KTNO/rGO-12 and KTNO collected between 0.1 and 1 mV s^−1^ (Figs. 4e and S26). Utilising Eq. S2, the value of *b* can be determined through a linear fit of the log of the peak current *i*_*p*_ and the scan rate *ν*, with a value of 0.5 indicating diffusion dominated and a value of 1 indicating capacitive dominated. For KTNO/rGO-12, the *b* value was evaluated to be 0.69, indicating that the charge storage process is majority intercalation, (Fig. 5f). In comparison, the *b* value for KTNO was highly capacitive at 0.93, presumably due to the K^+^ absorption on the surface of the KTNO particles. The inclusion of rGO is demonstrated to improve the diffusion of K^+^ into the KTNO nanoparticles and thus the K^+^ intercalation ability of KTNO, as a decrease in the *b* value is associated with an increase in intercalation [61].

**Fig. 6:**
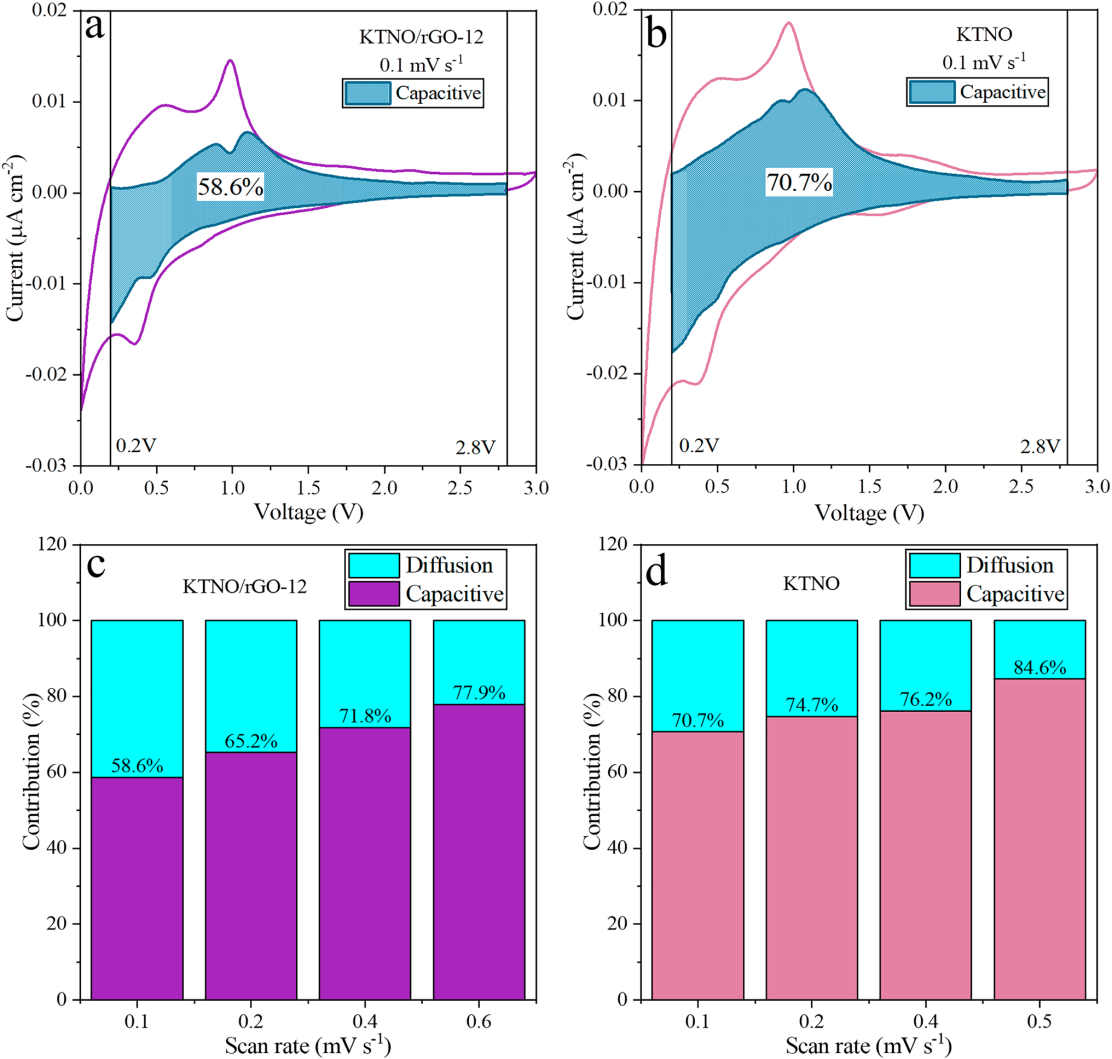
Analysis of the capacitive contribution to the observed current, with the turquoise area representing the capacitive charge storage. **a** Capacitive contribution for KTNO/rGO-12 at 0.1 mV s^−1^. **b** Capacitive contribution for KTNO at 0.1 mV s^−1^. **c** The ratio between the capacitive and diffusion contribution for KTNO/rGO-12 at different scan rates. **d** The ratio between the capacitive and diffusion capacitive contribution for KTNO at different scan rates

Utilising Dunn’s method, the ratio between the capacitive and diffusion contributions can be investigated for KTNO and KTNO/rGO-12 using Eq. S3 [62], to further highlight the effects of the rGO on the performance of the nanocomposite. Analysing the scans from 0.1–0.6 to 0.1–0.5 mV s^−1^ for KTNO/rGO-12 and KTNO, respectively, with boundaries set as 0.2–2.8 V allowed the capacitive ratio to be calculated, with the results graphically represented as a shaded area (Fig. 6a, b). The ratios for KTNO/rGO-12 were determined to be 58.6%, 65.2%, 71.8%, and 77.9% at scan rates 0.1, 0.2, 0.4, and 0.6 mV s^−1^ whereas the ratios for KTNO were 70.7%, 74.7%, 76.2%, and 84.6% (Fig. 6c, d) at scan rates of 0.1, 0.2, 0.4, and 0.5, respectively. These results show that the addition of rGO results in a lower capacitive contribution at low scan rates compared to KTNO without rGO, corroborating the *b* value obtained. The rGO undoubtably contributes to the increase in capacity observed, due to its high capacity as a KIB anode. However, as can be observed from the *b* value measurements and the capacitive contributions, there was an increase in K^+^ diffusion dominated processes and therefore a corresponding increase in K^+^ storage within KTNO, indicating that the rGO synergistically increases both electronic conductivity and K^+^ diffusion within KTNO.

To further explore the beneficial properties of KTNO/rGO nanocomposite, the mechanism of charge storage in KTNO/rGO-8 was investigated by in-operando powder XRD and supplemented by ex-situ XRD. This nanocomposite was chosen as it contained the least amount of carbon, allowing for higher quality patterns to be obtained with lower loading of the active material and at the same time, with a lower content of rGO. The scans were collected from 2 to 32° while discharging to 0.001 V and charging to 3 V (Fig. 6a). At the open circuit potential (OCP), the peaks corresponding to the (002), (102), (011), (200), (113), and (106) planes were clearly detected, giving a unit cell volume of 451.1 Å^3^. Upon K^+^ insertion, no noticeable peak shifts or intensity changes were observed from OCP to full discharge. The unit cell volume change is therefore minimal. With K^+^ extraction, no peak position changes were observed. These results indicate that the KTNO/rGO nanocomposite could be a low-strain material. This was supported by the ex-situ XRD patterns at the fully discharged and charged states, where no peak shifts or intensity changes were observed either (Fig. S28), removing possibility of the in-situ setup affecting the result. This suggests that within its current capacity, the KTNO/rGO nanocomposite can store charge with minimal volume change of the lattice, a significant benefit for long term stability (shown in Fig. 4c) as volume changes are associated with electrode disintegration and loss of capacity.

**Fig. 7:**
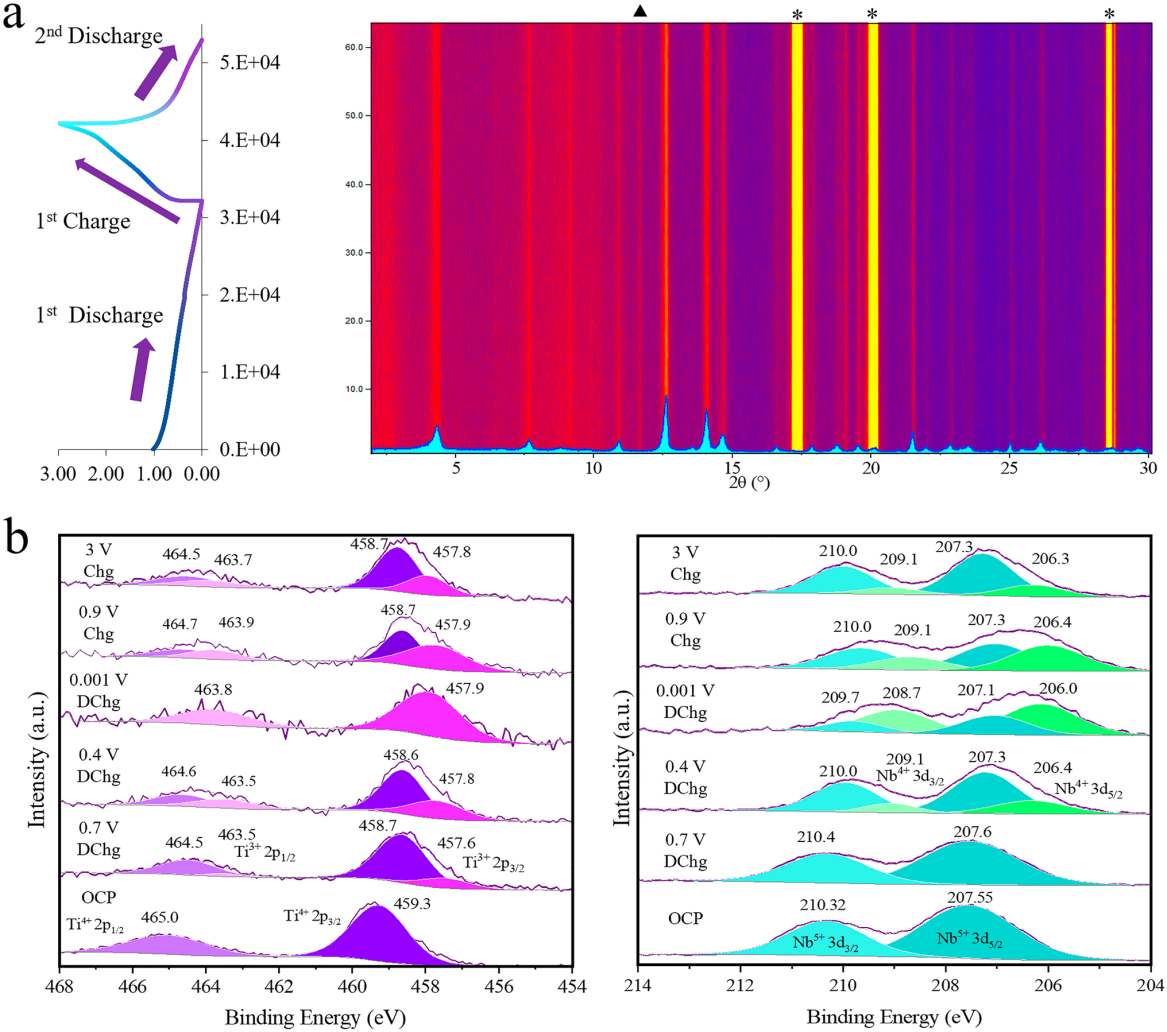
Mechanistic analysis of charge storage. **a** In-situ XRD of KTNO/rGO-8 during the charge–discharge process with inset of pristine KTNO/rGO-8 XRD pattern (*: Al; ▲: unidentified). **b** Ex-situ high resolution XPS spectra of Ti and Nb core lines at selected points along the charge–discharge curve

To investigate the mechanism of intercalation and the redox active metal centres, XPS analysis was performed on electrodes (Fig. 6b). By comparing the binding energies of the core electrons within the elements of the active material under different states of charge. At the open circuit potential (OCP), the high-resolution spectra could be well assigned with both Ti and Nb fully oxidised as their + 4 and + 5 states, respectively. However, when the voltage is decreased to 0.7 V, directly before the plateau, a contribution of Ti^3+^ was required to accurately fit the Ti spectrum, at 457.6 and 463.5 eV for the 2p_3/2_ and the 2p_1/2_ peaks, respectively. This suggests that the Ti^4+^/Ti^3+^ redox pair is partially activated before the plateau at 0.5 V and is in line with other literature results, as CVs for Ti-based materials in KIBs often do not have sharp peaks at well-defined voltages [21, 23, 53, 54]. The Nb showed no reduction at 0.7 V. At 0.4 V there was a clear indication that the Ti was reduced more significantly, and the Nb was starting to be reduced, implying the plateau arises mainly from the Nb redox pair Nb^5+^/Nb^4+^, with the Ti^4+^/Ti^3+^ contributing to it and across the whole voltage range. At full discharge, the Ti^4+^ contribution fully disappeared indicating that it was fully reduced via the intercalation of K^+^. The Nb however, showed the coexistence of Nb^5+^ and Nb^4+^, meaning it was not able to be fully reduced. Further evidence that the plateau is from the Nb redox pair, is that when charging to directly before the plateau at 0.9 V, the proportion of Nb^4+^ to Nb^5+^ changes less significantly that the Ti^4+^/Ti^3+^. Only after the plateau does the Nb significantly oxidise. Finally, full charging to 3 V still resulting in the contributions from Ti^3+^ and Nb^4+^ indicating that not all the intercalated K^+^ is able to de-intercalate, with this partial irreversibility being previously reported [19, 22, 34]. This is likely a contributor of the discrepancy between the theoretical capacity and the observed capacity, as a partial re-oxidation will leave K^+^ remaining in the structure, rendering it electrochemically inactive. Overall, these results show that both Ti and Nb are redox active and are contributing to the capacity of the KTNO (Fig. 7).

## Conclusions

To conclude, a KTNO/rGO-12 nanocomposite was successfully synthesised via solvothermal synthesis containing 12 wt% of rGO. It was then tested for its potassium storage properties as an anode material, achieving a first charge capacity of 128.1 mAh g^−1^ and second discharge capacity of 125.7 mAh g^−1^ at 20 mA g^−1^. The nanocomposite also showed excellent stability over 500 cycles, retaining 76.1% of the initial charge capacity. The rate performance was then tested up to a maximum current density of 1 A g^−1^, retaining 54.2 mAh g^−1^. The mechanism of charge storage was investigated via in-operando XRD with supplementary ex-situ XRD, indicating a low-strain material. This is an interesting result, as low-strain materials could have wide application in the energy storage industry, as structural breakdown could be minimised and cycling performance could be radically improved. Further ex-situ XPS analysis indicates that both Ti and Nb are redox active and are able to be fully reduced to Ti^3+^ and partially reduced to Nb^4+^. Within the wider context of the literature, KTNO represents an important continuation of investigation into promising alternatives to graphite and has proven to be both capable and sustainable KIB anode material and merits further investigation into its low-strain nature.

## Supplementary Information

Below is the link to the electronic supplementary material.

Supplementary file1 (PDF 1959 kb)
