# Supplementary material for: Layered Potassium Titanium Niobate/Reduced Graphene Oxide Nanocomposite as a Potassium-Ion Battery Anode
Source: Nanomicro Lett. 2023 Nov 6;16:1. doi: 10.1007/s40820-023-01222-2 (PMC10628103; doi:10.1007/s40820-023-01222-2)
Supplement: Supplementary file 1 — Supplementary file1 (PDF 1959 kb) [file 40820_2023_1222_MOESM1_ESM.pdf]

Supplementary Information for

Layered Potassium Titanium Niobate/Reduced Graphene Oxide Nanocomposite as a Potassium-Ion Battery Anode

Charlie A. F. Nason[1], Ajay Piriya Vijaya Kumar Saroja[1], Yi Lu[1], Runzhe Wei[1], Yupei Han[1], Yang Xu[1,] *

[1] Department of Chemistry, University College London, 20 Gordon Street, London WC1H 0AJ, UK

*Corresponding author. E-mail: y.xu.1@ucl.ac.uk (Yang Xu)

Supplementary Figures and Tables

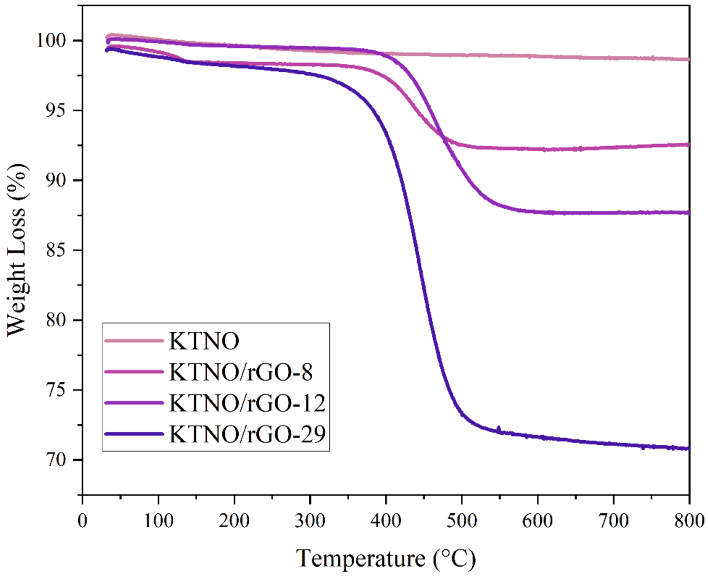

Fig. S1 TGA results of KTNO/rGO-8, KTNO/rGO-12 and KTNO/rGO-29

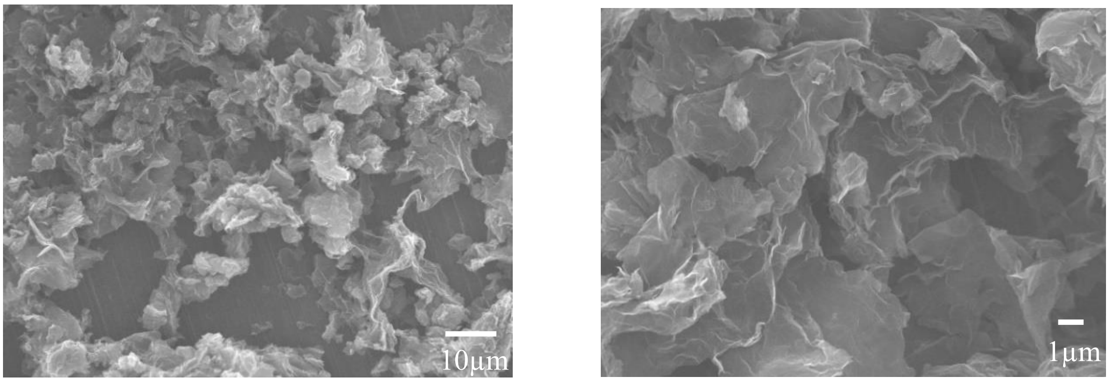

Fig. S2 SEM images of rGO

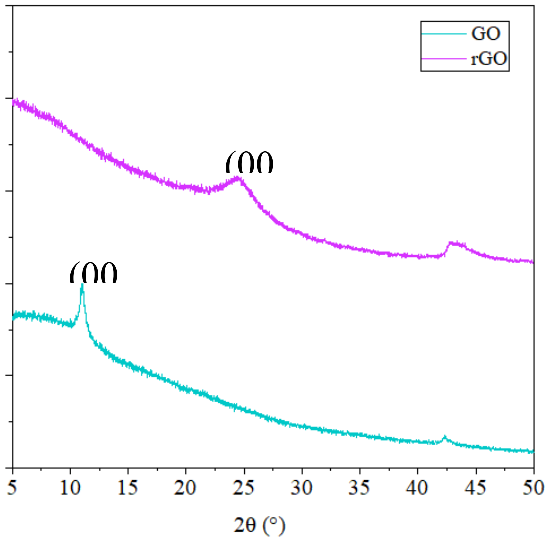

Fig. S3 XRD pattern of GO and rGO

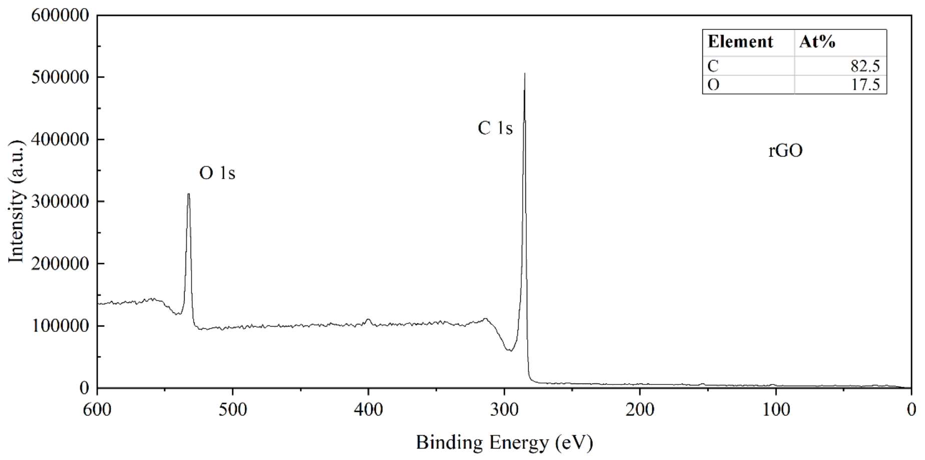

Fig. S4 XPS survey spectra of rGO

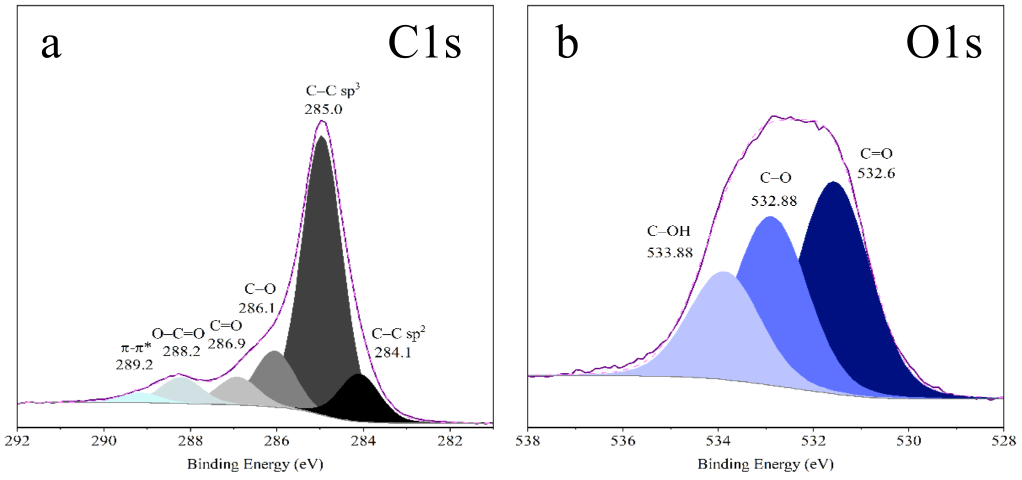

Fig. S5 High resolution XPS spectra of rGO. **a** C 1s **b** O 1s

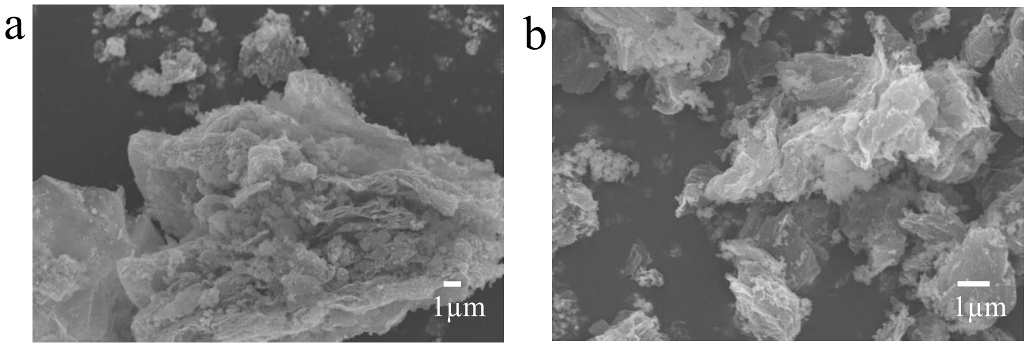

Fig. S6 SEM images of **a** KTNO/rGO-8. **b** KTNO/rGO-29

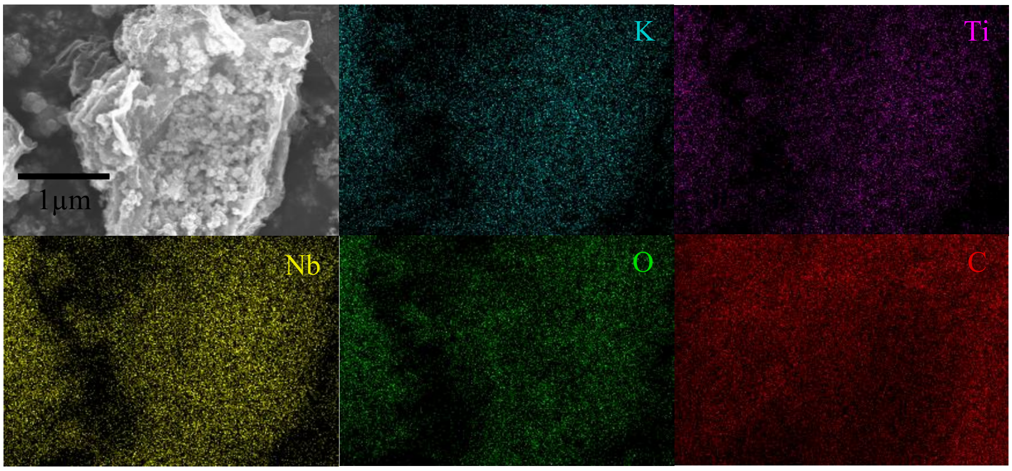

Fig. S7 EDS mapping of KTNO/rGO-8

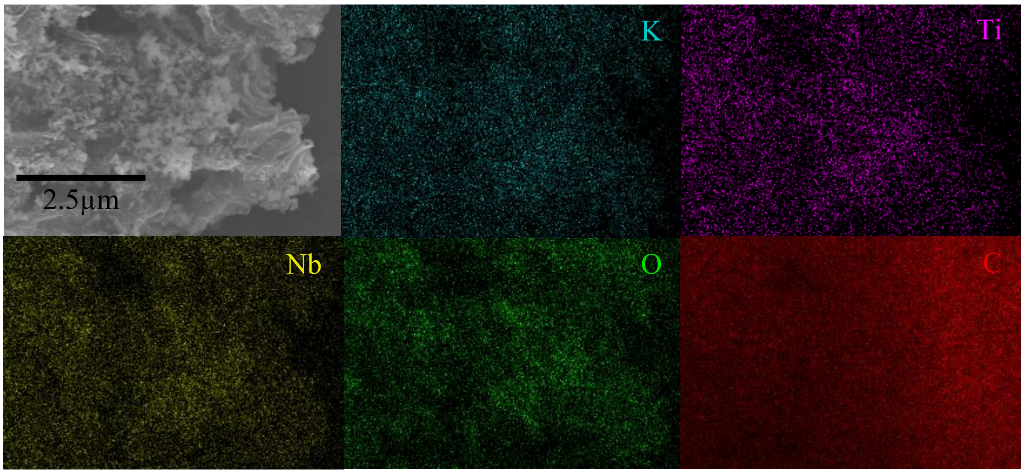

Fig. S8 EDS mapping of KTNO/rGO-29

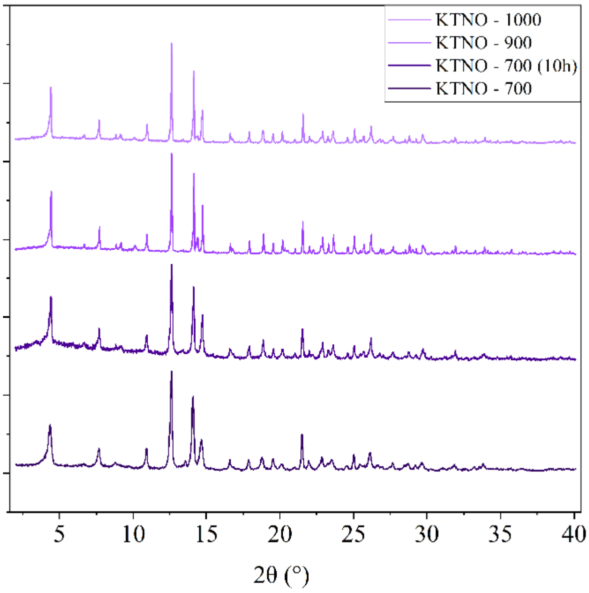

Fig. S9 XRD patterns of KTNO-700, KTNO-700-10h, KTNO-900 and KTNO-1000

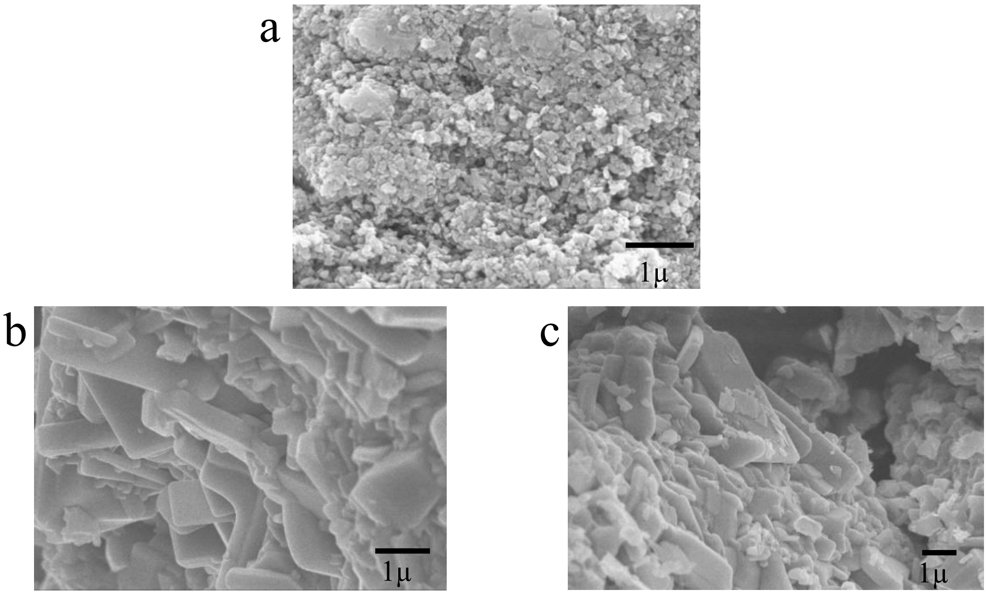

Fig. S10 SEM images of **a** KTNO-700. **b** KTNO-900. **c** KTNO-1000

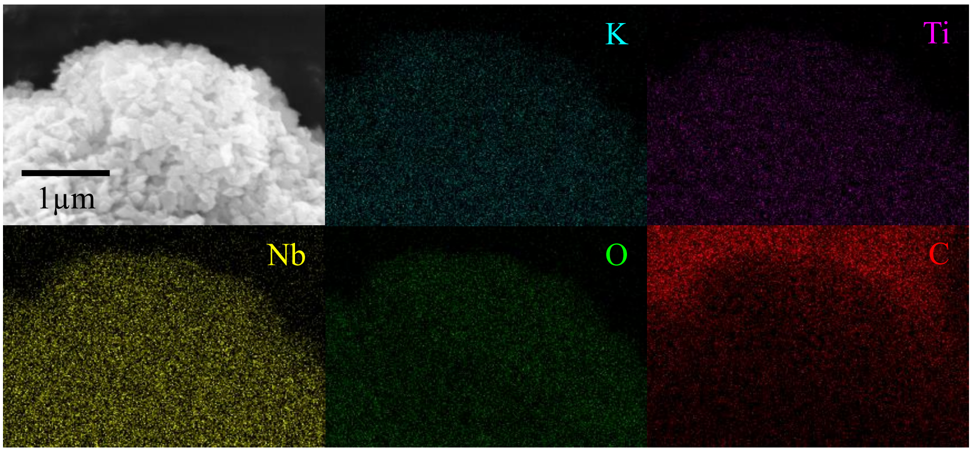

Fig. S11 EDS mapping of KTNO

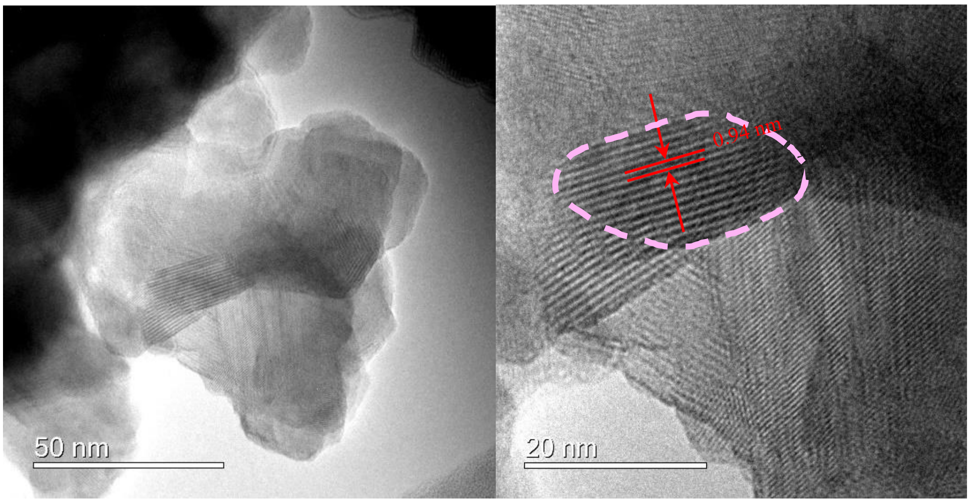

Fig. S12 TEM and HRTEM image of KTNO

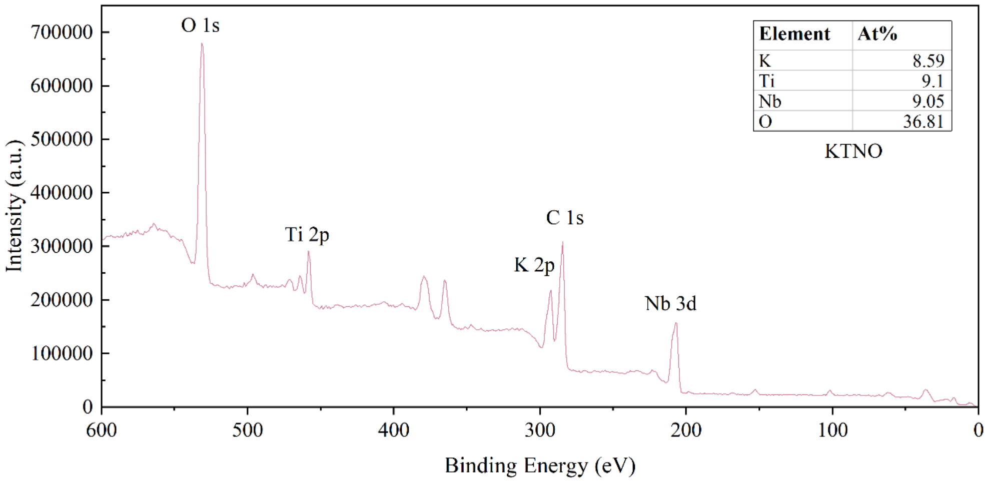

Fig. S13 XPS survey spectra of KTNO

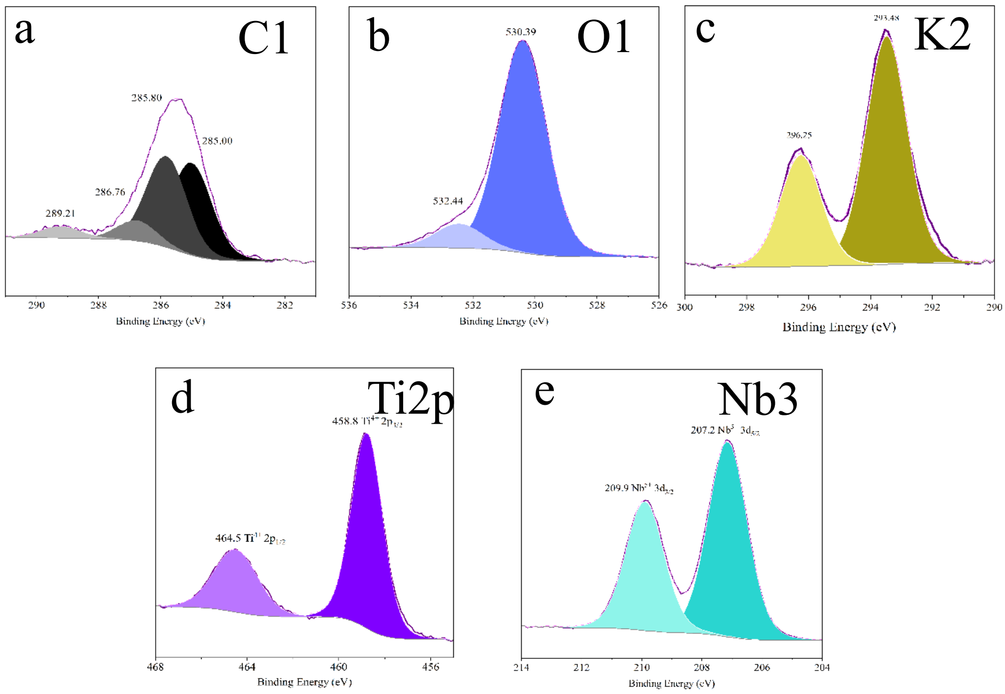

Fig. S14 High resolution XPS spectra of KTNO **a** C1s. **b** O1s. **c** K2p. **d** Ti. **e** Nb

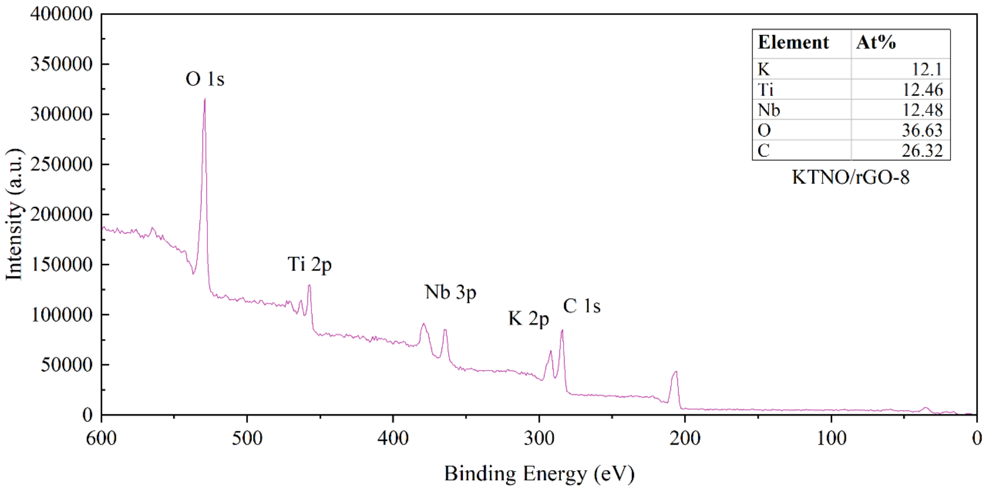

Fig. S15 XPS survey spectra of KTNO/rGO-8

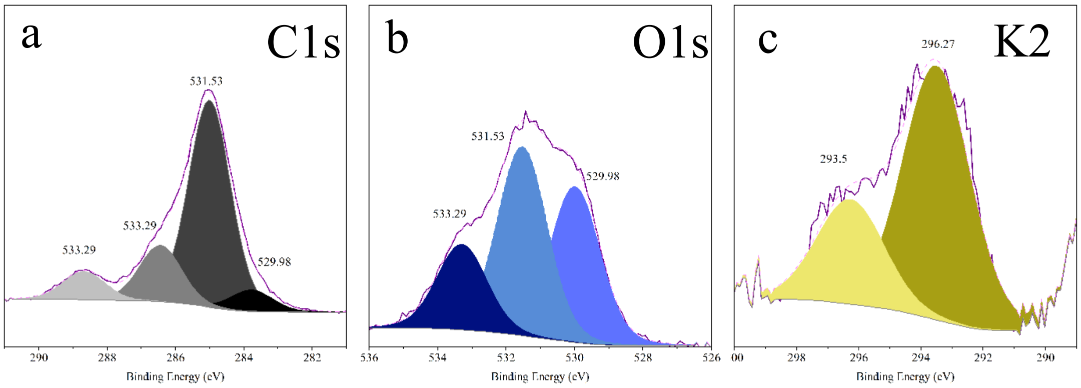

Fig. S16 High resolution XPS spectra of KTNO/rGO-12 **a** C1s. **b** O1s. **c** K2p

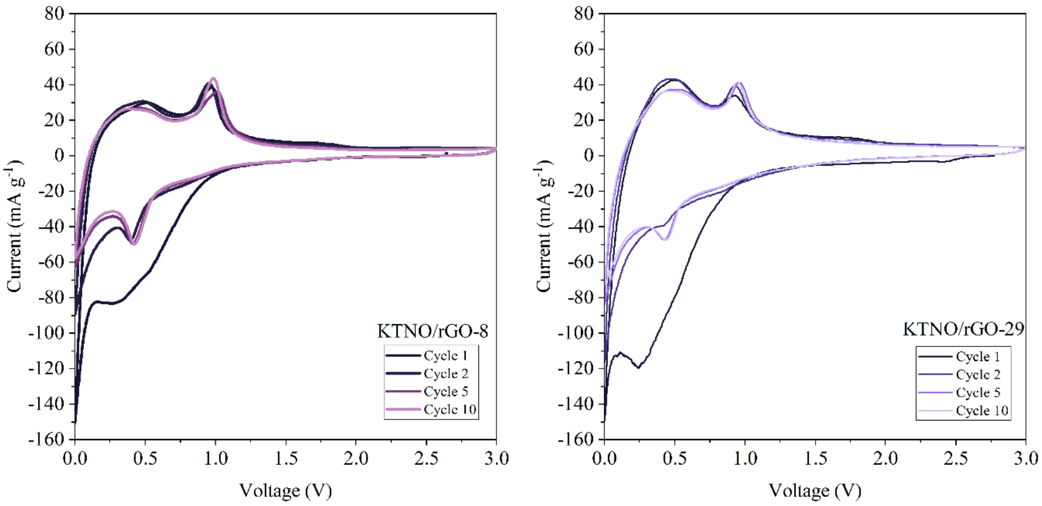

Fig. S17 CV curves for KTNO/rGO-8 and KTNO/rGO-29

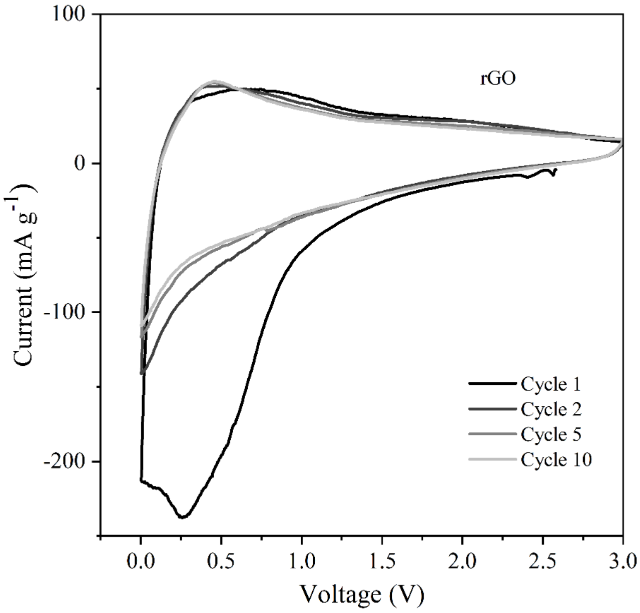

Fig. S18 CV curves for rGO

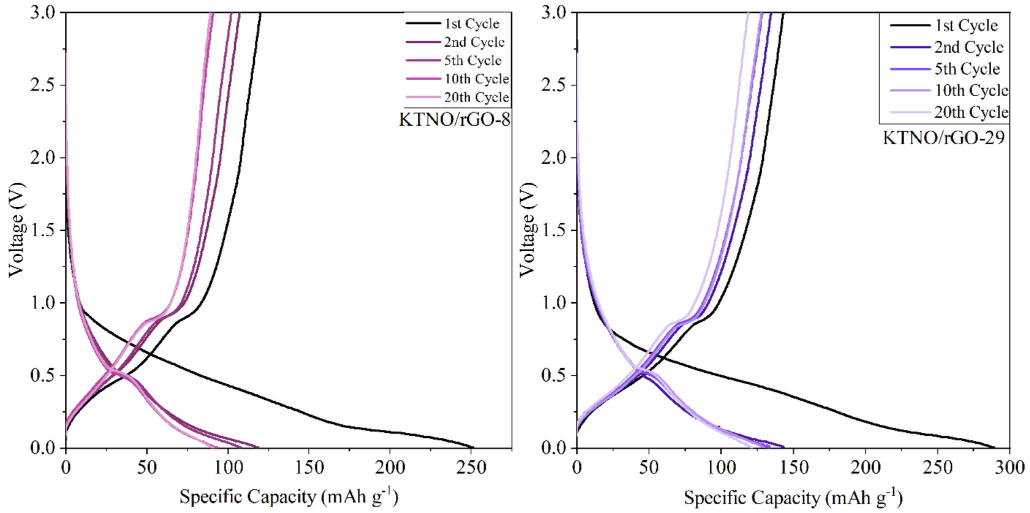

Fig. S19 Galvanostatic charge-discharge curves of KTNO/rGO-8 and KTNO/rGO-29 at 20 mA g^{-1}

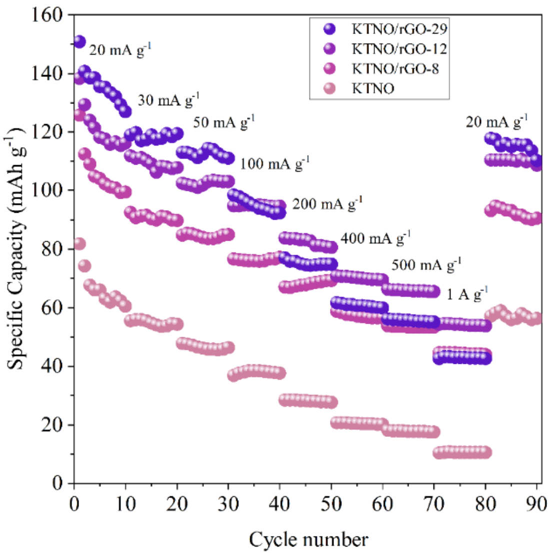

Fig. S20 Rate performance of KTNO, KTNO/rGO-8, KTNO/rGO-12 and KTNO/rGO-29

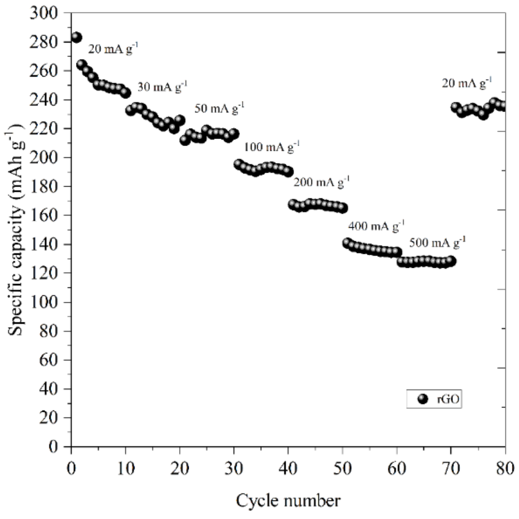

Fig. S21 Rate performance of rGO

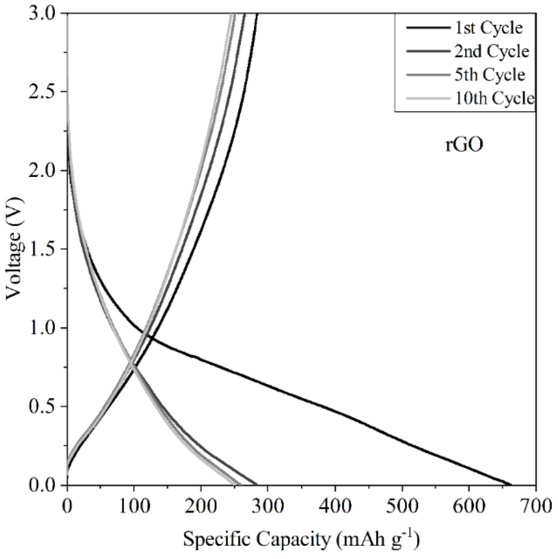

Fig. S22 GCD curves of rGO

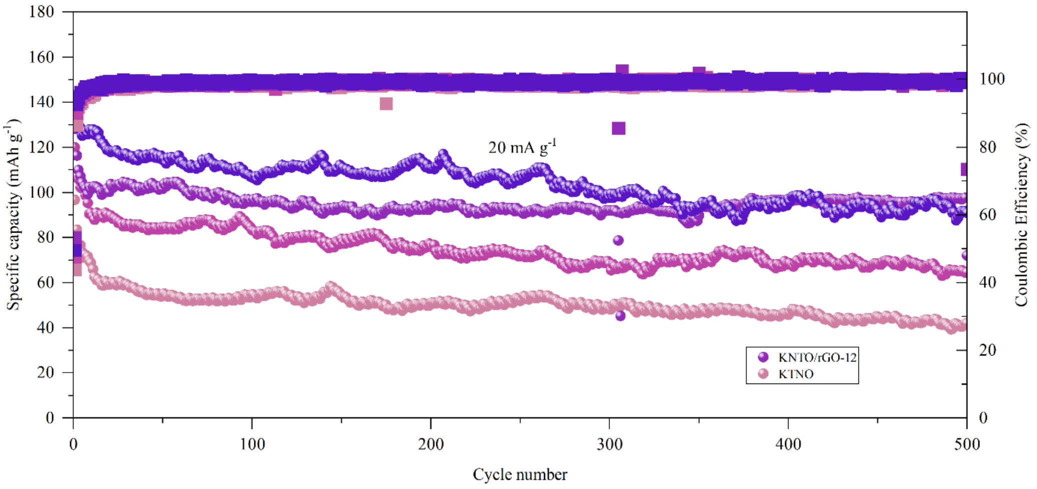

Fig. S23 Cycling performance of KTNO/rGO and KTNO

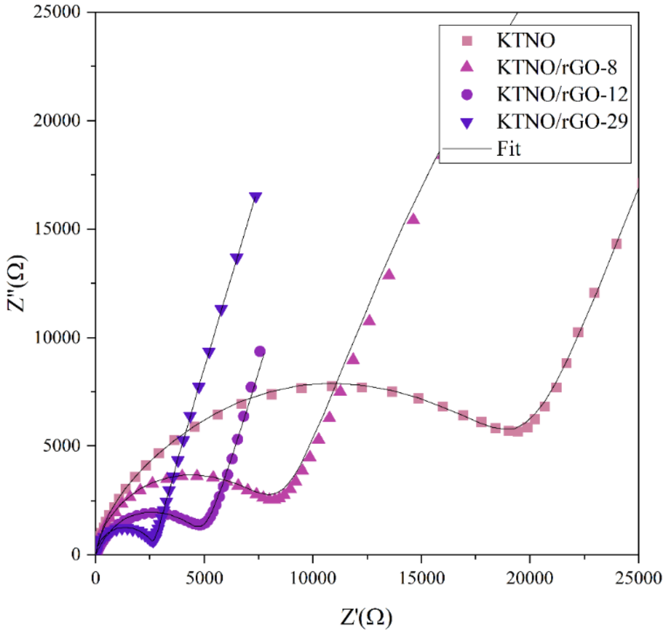

Fig. S24 EIS results for KTNO and the nanocomposites KTNO/rGO-8, KTNO/rGO-12 and KTNO/rGO-29

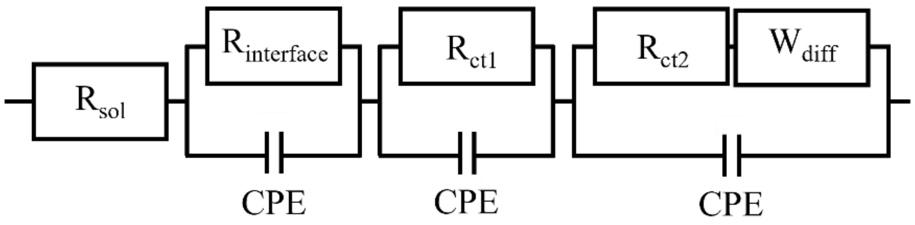

Fig. S25 Equivalent circuit used for fitting.

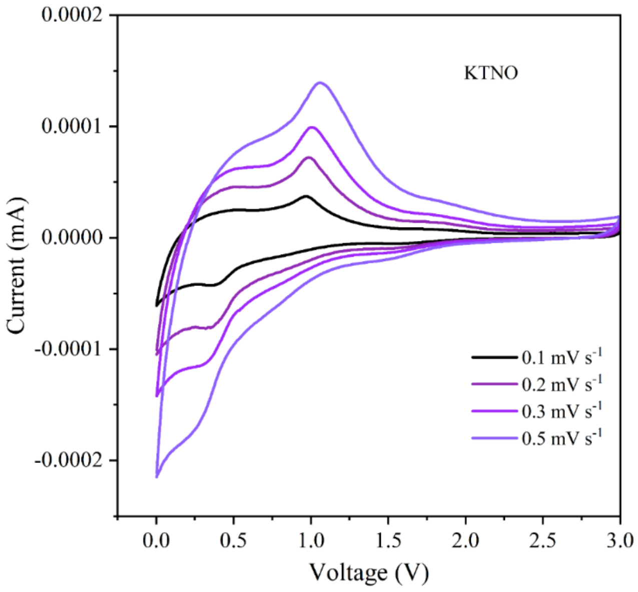

Fig. S26 CV curves of KTNO at scan rates from 0.1 – 0.5 mV s^{-1}

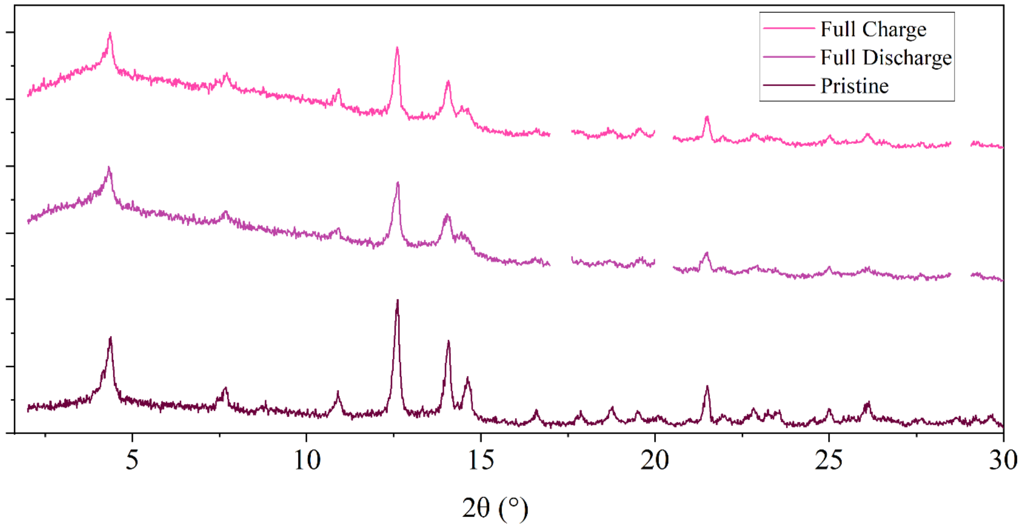

Fig. S27 Ex-situ XRD of KTNO/rGO-8. Al peaks removed for clarity

Equation S1:

$$D = \frac{4}{9\pi} \cdot \left(\frac{E_4 - E_0}{E_2 - E_0}\right)^2 \cdot \frac{{r_p}^2}{t_p}$$

Equation S2:

$$i_p = a\nu^b$$

Equation S3:

$$i(V) = k_1\nu + k_2\nu^{1/2}$$
